# Systematic review and meta‐analysis evaluating the effects electric bikes have on physiological parameters

**DOI:** 10.1111/sms.14155

**Published:** 2022-03-23

**Authors:** Jenna McVicar, Michelle A. Keske, Reza Daryabeygi‐Khotbehsara, Andrew C. Betik, Lewan Parker, Ralph Maddison

**Affiliations:** ^1^ Institute for Physical Activity and Nutrition Deakin University Geelong Victoria Australia

**Keywords:** electric bike, energy expenditure, heart rate, human physiology, metabolic equivalents, oxygen uptake, Physical activity, power output

## Abstract

**Background:**

There is a universal need to increase the number of adults meeting physical activity (PA) recommendations to help improve health. In recent years, electrically assisted bicycles (e‐bikes) have emerged as a promising method for supporting people to initiate and maintain physical activity levels. To the best of our knowledge, there have been no meta‐analyses conducted to quantify the difference in physiological responses between e‐cycling with electrical assistance, e‐cycling without assistance, conventional cycling, and walking.

**Methods:**

A systematic review and meta‐analysis was conducted following PRISMA guidelines. We identified short‐term e‐bike studies, which utilized a crossover design comparing physiological outcomes when e‐cycling with electrical assistance, e‐cycling without electrical assistance, conventional cycling, or walking. Energy expenditure (EE), heart rate (HR), oxygen consumption (VO_2_), power output (PO), and metabolic equivalents (METs) outcomes were included within the meta‐analysis.

**Results:**

Fourteen studies met our inclusion criteria (*N* = 239). E‐cycling with electrical assistance resulted in a lower energy expenditure (EE) [SMD = −0.46 (−0.98, 0.06), *p* = 0.08], heart rate (HR) [MD = −11.41 (−17.15, −5.68), *p* < 0.000, beats per minute], oxygen uptake (VO_2_) [SMD = −0.57 (−0.96, −0.17), *p* = 0.005], power output (PO) [MD = −31.19 (−47.19 to −15.18), *p* = 0.000, Watts], and metabolic equivalent (MET) response [MD = −0.83 (−1.52, −0.14), *p* = 0.02, METs], compared with conventional cycling. E‐cycling with moderate electrical assistance resulted in a greater HR response [MD 10.38 (−1.48, 22.23) *p* = 0.09, beats per minute], and VO_2_ response [SMD 0.34 (−0.14, 0.82) *p* = 0.16] compared with walking.

**Conclusions:**

E‐cycling was associated with increased physiological responses that can confer health benefits.

## 1 BACKGROUND

Regular participation in physical activity (PA) is associated with numerous health benefits including the prevention of cardiovascular disease and type 2 diabetes,^1^ yet over one quarter of adults worldwide are physically inactive.^2^ The World Health Organization (WHO) estimates 27.5% of adults do not meet the recommended level of PA and have subsequently highlighted the need for PA promotion.^2^ There is a universal need to increase the number of adults meeting PA recommendations to help improve health on an individual level and to help ease the burden on healthcare systems.^3^

The WHO recommends all adults should undertake 150–300 min of moderate intensity or 75–150 min of vigorous activity per week or some equivalent combination.^2^ The most frequently cited reason for not being physically active by individuals is lack of time;^4^ therefore, plans and strategies to promote physical activity levels should entail people implementing activity in their everyday life. Walking and cycling for short journeys (termed active transport) has been shown to be effective for achieving recommended levels of PA;^5^ however, cycling for transport accounts for 1% of trips in the USA, Canada, and Australia.^6^ In recent years, electrically assisted bicycles (e‐bikes) have emerged as a promising method for supporting people to increase PA levels via active transport.^7^ Compared with conventional cycling, e‐bikes help individuals to cycle further and for longer periods of time.^8^ E‐bikes also offer an ideal opportunity to encourage individuals to include PA into their daily lives by replacing short car trips with cycling.

A 2018 systematic review of 17 studies (*N* = 300) found e‐cycling was associated with improved cardiorespiratory fitness and increased PA levels.^9^ A 4‐week e‐cycling intervention found e‐bike use increased cardiorespiratory fitness levels and reduced 2‐h post oral glucose tolerance test blood glucose levels.^10^ Furthermore, another 4‐week e‐bike intervention found e‐cycling increased power output. E‐cycling was also associated with feelings of enjoyment among those who were physically inactive.^10^ While short‐term studies assessed in the systematic review reported on the acute physiological effects of e‐cycling compared with conventional cycling and walking, no meta‐analysis was conducted to quantify the magnitude of effect on physiological parameters. This study extends upon this previous systematic review by updating the literature and conducting a meta‐analysis to determine the magnitude of physiological response elicited by short‐term e‐cycling. In doing so, we provide much needed data on the physiological responses associated with e‐cycling, which will help inform future programs to promote e‐cycling and associated health benefits.

## 2 MATERIALS AND METHODS

This systematic review and meta‐analysis were conducted in accordance with the Preferred Reporting Items for Systematic Reviews and Meta‐analyses (PRISMA) statement (see Appendix S2 for flow diagram) and was registered with PROSPERO [CRD42020203905]. For this review, e‐bikes were defined as electrically assisted bicycles requiring the rider to pedal for assistance to be provided.

Inclusion criteria included acute, single ride comparison studies which assessed e‐cycling with assistance compared to e‐cycling without assistance, conventional cycling or walking. Acute e‐bike studies predominantly measure energy expenditure (EE), heart rate (HR), oxygen uptake (VO_2_), power output (PO) and report metabolic equivalents (METs). As such, studies which measured these physiological responses were included within this systematic review and meta‐analysis. Experimental or observational studies, pre‐ and post‐design, quasi experimental, randomized, and non‐randomized cross‐over trials were included. Only papers written in English were included. Assessment of outcomes was laboratory or field based. Studies with adults, adolescents, and children were included.

Exclusion criteria included studies without a comparison or control group, studies that examined environmental effects of e‐bike use, and long‐term studies (≥4‐week intervention period).

Search strategy: Databases searched were PsychINFO, MEDLINE, Embase, ISI Web of Science, CINAHL complete, SPORTDiscuss, Scopus, and PubMed, and were searched from database inception to August 2020. Our full search strategy is available in an additional word document (see Appendix S1).

Researchers (JM and MK) independently screened and reviewed titles and abstracts to identify eligible studies. Researchers (JM, AB, and LP) assessed full‐text articles for eligibility. Researchers (JM and RD) independently performed data extraction and quality assessment for the studies included within the meta‐analysis. Any discrepancies were discussed with RM.

Quality assessment: The quality assessment tool for quantitative studies developed by the Effective Public Health Practice Project^11^ was used to assess the overall rating of included studies according to the following criteria: (1) selection bias, (2) study design, (3) control of confounders, (4) blinding, (5) reliability and validity, and (6) withdrawals and dropout. Studies were rated as strong, moderate, or weak across each criterion, which provided an overall rating of quality. This quality assessment tool is recommended for non‐RCT studies.^12^

Data extraction: JM and RD extracted data from the papers independently. If papers did not include a full data set, the authors were contacted. Data were extracted using Covidence systematic review software (Veritas Health Innovation, Melbourne, Victoria, Australia), and discrepancies were discussed between researchers. Data extracted included authors and their institution, methodological design, participant population and characteristics, intervention characteristics, and intervention outcomes. Data were synthesized and presented in table format with narrative description.

## 3 SUMMARY MEASURES

Data extracted from the studies were continuous outcomes; therefore, within the meta‐analysis, these data were assessed as either mean difference or standardized mean difference (SMD). EE (kcal) data are presented as SMD. HR data are reported as mean difference as all studies included within the meta‐analysis reported HR in beats per minute (BPM). VO_2_ (L/min or ml/kg/min) is reported as SMD. PO is reported as mean difference as all studies reported PO in watts (W). METs are reported as mean difference. Nine studies reported METs, four studies were included within the MET meta‐analysis and are reported as mean difference. All studies which reported METs are described narratively. For comparisons of SMD, the value was reported as units of standard deviation rather than the units reported for the outcome measured. We considered a SMD of 0.2 as a small effect, 0.5 as a moderate effect, and 0.8 as a large effect.^13^

## 4 RESULTS

A total of 811 articles were obtained from the initial search and one paper was found through hand searching. After removal of 184 duplicates, 628 studies were title and abstract screened. From the 628 identified, 39 full texts were reviewed. After full‐text review, 14 studies were identified with 12 providing complete data, an additional word document containing the PRISMA flow diagram is available (see Appendix S3). One author did not respond to our request for further information and one author was unable to follow‐up on our request; thus, 12 studies were included in the meta‐analysis and results for the 14 included studies are presented narratively.

Characteristics of all included studies are presented in Table 1. Of the 14 included studies, six assessed EE,^14^, ^15^, ^16^, ^17^, ^18^, ^19^ 12 assessed HR^14^, ^16^, ^17^, ^18^, ^19^, ^20^, ^21^, ^22^, ^23^, ^24^, ^25^, ^26^ five assessed PO,^14^, ^16^, ^17^, ^18^, ^20^ eight assessed VO_2_, ^15^, ^16^, ^17^, ^19^, ^20^, ^24^, ^25^, ^27^ and nine reported METs.^15^, ^16^, ^17^, ^18^, ^19^, ^20^, ^21^, ^24^, ^27^ All studies utilized a crossover design. Eight studies were conducted in Europe (two in France, two in Germany, and one in Norway, Switzerland, The Netherlands, and Belgium), five in the USA, and one in Australia. Sample sizes ranged from 3 to 33. Most studies recruited adults who were healthy, two studies recruited adults who were sedentary^20^, ^24^ and one study recruited people with coronary artery disease.^15^ Six studies compared e‐cycling (with electrical assistance) to conventional cycling,^15^, ^19^, ^20^, ^22^, ^25^, ^27^ five studies compared e‐cycling with no electrical assistance with e‐cycling with electrical assistance.^14^, ^17^, ^18^, ^21^, ^26^ Two studies compared e‐cycling with electrical assistance to conventional cycling and walking.^16^, ^24^ Timing between trials ranged from 2 min to 1‐week. Distances rode on the bikes ranged from 46m circuits to 16 km routes. Some studies included varying topography^15^, ^16^, ^20^, ^21^, ^23^, ^26^, ^27^ where others maintained a flat route.^14^, ^18^, ^19^, ^22^ Throughout many of the studies, participants were advised to cycle at a self‐selected pace. Only one study^17^ pre‐specified target speeds, 16 km/h and 21 km/h; this study also included a ride, which allowed participants to e‐cycle at a self‐selected pace.

**TABLE 1:** Study design and participant characteristics of included studies.

First author, year	Study design	Country	Participants	Participant characteristics	Physiological parameter	Intervention characteristics (What was compared?)	Ride characteristics
Alessio, H. 2021	Randomized crossover	USA	Total 30: Age (years) 26.2 ± 12.7 Height (m) 1.8 ± 0.1 Body weight (kg) 77.6 ± 18.4 BMI (kg/m^2^): 25.1 ± 4.2	Healthy adults	Energy expenditure (kcal/hr), % HR max, % VO_2_ max	E‐bike to conventional bike	E‐bike low assist v e‐bike moderate assist v conventional bike
Berntsen, S. 2017	Randomized crossover	Norway	Total 8: 23–54 years old.	Healthy adults	VO_2_ (%)	E‐bike to conventional bike	E‐bike assistance self‐selected v conventional bike
Bini, R. 2019	Randomized crossover	Australia	Total 20: Age (years) 40 ± 15 Height (cm) 177 ± 8 Body weight (kg) 78 ± 11	10 postal workers, 10 recreational cyclists	Energy expenditure (kcal), heart rate (bpm), and power output (W)	E‐bike with various electrical assistance	E‐bike no electrical assistance v e‐bike with electrical assistance
Gojanovic, B. 2011	Crossover	Switzerland	Total 18: Age (years): 35.7 ± 9.7 Height (m): 1.70 ± 0.09 BMI (kg/m^2^): 24.0 ± 3.3 Body weight (kg): 70.1 ± 13.8	Sedentary adults	Heart rate, VO_2_ (l/min)	E‐bike was compared with conventional bike and walking	E‐bike high assistance v e‐bike standard assistance v conventional bike v walking
Hall, C. 2019	Convergent mixed methods approach	USA	Total 33: Average age: just under 38 years old.	Experienced mountain bikers	Heart rate (bpm)	E‐mountain bike compared with conventional mountain bike	E‐bike assistance not advised v conventional mountain bike
Hansen, D. 2018	Randomized crossover clinical trial	Belgium	Total 15: Patients with CAD. Age (years) 64 ± 7	Coronary artery disease patients	Energy expenditure (kcal), Mean VO_2_ (ml/min)	e‐bike v conventional bike	E‐bike low assistance v e‐bike high assistance v conventional bike
Hoj, T. 2018	Crossover	USA	33 participants, average age 22 years old.	Healthy adults	Heart rate average (bpm), heart rate max (bpm)	e‐bike v conventional bike	E‐bike assistance level not available v conventional bike
Langford, C. 2017	Semi‐crossover	USA	6 females, 11 males. BMI (kg/m^2^): females – 23.1, males – 26.1.	Healthy adults	Energy expenditure (kcal), heart rate (bpm), power output (W), VO_2_	E‐bike was compared to conventional bike and walking	E‐bike high electrical assistance v conventional bike v walking
LaSalle, D. 2017	Crossover	USA	Total 12: Females (mean ± SE): Age (years) 22 ± 1 Height (cm) 171 ± 2 Weight (kg) 71.2 ± 5 Body fat (%) 23.4 ± 3.3 Males (mean ± SE): Age (years) 25 ± 1 Height (cm) 177 ± 2 Weight (kg) 87.9 ± 6 Body fat (%) 16.8 ± 1.9	Healthy active adults	Heart rate max (%), VO_2_ max (%)	E‐bike with various levels of electrical assistance	E‐bike with pedal assist mode v e‐bike without electrical assistance
Louis, J. 2012	Randomized crossover	France	Total 20: Two participant groups: Trained v untrained. Trained: Age (years) 38.7 ± 14.8, Height (m) 1.77 ± 0.06, Body weight (kg) 69.2 ± 5.8 BMI (kg/m^2^): 22.0 ± 1.1 Untrained: Age (years) 28.9 ± 6.3, Height (m) 1.72 ± 0.07, Body weight (kg) 66.1 ± 14.8, BMI (kg/m^2^): 22.2 ± 3.7	10 trained adults & 10 untrained adults	Energy expenditure (kcal), heart rate (bpm), power output (W), VO_2_ (ml/kg/min)	E‐bike with various assistance levels	E‐bike unassisted v e‐bike light electrical assistance v e‐bike high electrical assistance
Meyer, D. 2014	Crossover	Germany	Total 3 males: Age (years) 25–27, Weight (kg) 71–79, Height (cm) 176–183	Recreational cyclists	Lactate (mmol/L), heart rate (bpm), Borg scale.	E‐bike with and without electrical assistance	E‐bike with electrical assistance v e‐bike without electrical assistance
Simons, M. 2009	Crossover	The Netherlands	12 Total: Age (years): 52.2 ± 8.7 Height (cm) 173.3 ± 7.6 BMI (kg/m^2^): 24.5 ± 2.6 Body weight (kg): 73.6 ± 9.7	Habitually active adults – 7 met PA guidelines	Energy expenditure (kcal), Heart rate (bpm), Power output (W)	E‐bike with no support v e‐bike with varying electrical assistance	E‐bike No Electrical Assistance v E‐bike Eco Electrical Assistance v E‐bike Power Support
Sperlich, B. 2012	Randomized crossover	Germany	8 females, Age (years) 38 ± 15 Body mass (Kg) 71.3 ± 12.9 BMI (kg/m^2^): 25.3 ± 2.1	Sedentary adults	Heart rate (bpm), VO_2_ (ml.kg.min), Mean power output (W)	E‐bike v conventional bike	E‐bike with assistance v conventional bike
Theurel, J. 2012	Crossover	France	10 total: 5 females: Age (years) 30 ± 12 Height (cm) 163 ± 2 Weight (kg) 58 ± 4 5 Males: Age (years) 35 ± 14 Height (cm) 177 ± 9 Weight (kg) 73 ± 9	Healthy adults – moderate PA level	Heart rate (bpm) VO_2_ (ml.kg.min)	E‐bike v conventional bike	E‐bike assisted cycling v conventional bike

To provide context to the levels of electrical assistance e‐bikes provide, we reported the standards from Bosch, an e‐bike battery supplier. A low level of electrical assistance provided 40% support, moderate assistance provided 100% support, and a high level of assistance can range from 150% to 250% support. When e‐cycling with 100% support, the support provided would match that being produced by the individual cycling, for example 40 W output with 100% support would allow an 80W output, similarly 150% support on a 40 W e‐cycle would provide a total of 100 W output.

## 5 QUALITY ASSESSMENT

The quality assessment tool for quantitative studies was applied (Table 2) and resulted in one strong rating,^15^ one moderate rating,^19^ and 12 weak ratings.^14^, ^16^, ^17^, ^18^, ^20^, ^21^, ^24^, ^25^, ^26^, ^27^ Overall, the methods were rated as strong. Methods were assessed on validation and reliability of the data collection tools used. All included studies were rated as weak for blinding. Binding was not included in the global rating due to the nature of the methodologies (i.e., the inability to reliably blind participants to an e‐bike). Selection bias and confounders were generally rated as weak. Selection bias was rated on the likelihood participants were representative of the target population. Confounders were rated on the differences between study groups and indication of control for these differences. Studies were classified as strong if they received no weak rating, classified as moderate if they received one weak rating and classified as weak if they received two or more weak ratings. One study^15^ was powered to detect a significant difference in calorie expenditure between cycling conditions.

**TABLE 2:** Quality assessment tool for quantitative studies.

Study	Selection bias	Design	Confounders	Blinding	Methods	Drop‐outs	Global rating
Alessio, 2021	Strong	Moderate	Weak	Weak	Strong	Strong	Moderate
Berntsen, 2017	Weak	Moderate	Weak	Weak	Strong	Strong	Weak
Bini, 2019	Weak	Moderate	Weak	Weak	Strong	Strong	Weak
Gojanovic, 2011	Weak	Weak	Weak	Weak	Strong	Strong	Weak
Hall, 2019	Moderate	Weak	Weak	Weak	Strong	Weak	Weak
Hansen, 2018	Strong	Strong	Strong	Weak	Strong	Strong	Strong
Hoj, 2018	Moderate	Weak	Weak	Weak	Strong	Strong	Weak
LaSalle, 2017	Weak	Moderate	Weak	Weak	Strong	Strong	Weak
Langford, 2017	Weak	Moderate	Weak	Weak	Moderate	Strong	Weak
Louis, 2012	Weak	Moderate	Strong	Weak	Strong	Weak	Weak
Meyer, 2014	Weak	Weak	Moderate	Weak	Strong	Strong	Weak
Simons, 2009	Weak	Weak	Weak	Weak	Strong	Strong	Weak
Sperlich, 2012	Weak	Strong	Weak	Weak	Strong	Weak	Weak
Theurel, 2012	Weak	Weak	Weak	Weak	Strong	Moderate	Weak

Note

Strong = no weak rating; moderate = one weak rating; weak = two or more weak. ratings.

## 6 OUTCOME MEASURES

### 6.1 Narrative review – METs

Nine studies^15^, ^16^, ^17^, ^18^, ^19^, ^20^, ^21^, ^24^, ^27^ assessed METs with e‐cycling with electrical assistance compared with conventional cycling or e‐cycling without electrical assistance. Reporting of METs within the studies was inconsistent, many authors reported mean or range of MET values, many studies did not report mean and SD. Four studies were included within the meta‐analysis,^15^, ^19^, ^20^, ^24^ the included studies compared METs between e‐cycling with moderate assistance and conventional cycling. Two studies used walking as a comparator.^16^, ^24^ METs ranged from 3 to 10.9. Overall, e‐cycling with electrical assistance was associated with lower MET values compared with its control comparator; however, some e‐cycling was associated with moderate (3–6 METs)^16^, ^17^, ^18^, ^19^, ^20^ to vigorous (MET range >6 METS) intensity activity.^15^, ^21^, ^24^, ^27^ Louis et al^17^ only provided ranges for MET data and as such were excluded from Table 3. Louis et al^17^reported all participants cycled at an intensity of at least 6 METs with no electrical assistance. With electrical assistance, Louis et al^17^ reported untrained participants e‐cycled at intensity of >6 METs; a similar intensity was observed only in trained participants who cycled at 21km/h with moderate electrical assistance. They reported the highest level of electrical assistance was associated with 3 to 6 METs.^17^ Langford et al^16^ provided an average of the MET values from the three segments included within their studies cycle route, a breakdown of the MET values reported are specified in Table 3.

**TABLE 3:** Metabolic Equivalent (METs) means from included studies.

Study	Conventional cycling METs (mean)	E‐bike no electrical assistance METs (mean)	E‐bike moderate electrical assistance METs (mean)	E‐bike high electrical assistance METs (mean)	Walking METs (mean)
Alessio, 2021	6.7		5.8	4.8	
Berntsen, 2017	10.9^a^		8.5^a^		
Gojanovic, 2011	8.2		7.3	6.1	6.5
Hansen, 2018	6.4		6.6	6.0	
La Salle, 2017		8.5	8.3		
Langford, 2017 (downhill)	3.9		3.7		3.8
Langford, 2017 (flat)	5.2		4.5		4.1
Langford, 2017 (uphill)	7.6		6.6		5.3
Langford, 2017 (average)	5.8		5.1		4.8
Sperlich, 2012	7.1		5.2		
Simons, 2009		6.1	5.7	5.2	

Note

Average MET values of included studies only mean reported to allow for consistency between studies; SD not always reported by authors.

^a^ Authors reported median values.

### 6.2 Meta‐analysis–EE, HR, VO_2_, PO, and METs

Random effect meta‐analysis was conducted, heterogeneity was assessed by Chi‐square test and reported as *I* ^2^. E‐cycling with moderate assistance was compared with conventional cycling for all outcomes. E‐cycling with moderate assistance was compared with e‐cycling without assistance for EE, HR, VO_2_, and PO. E‐cycling with high assistance was compared with conventional cycling for HR and VO_2_ and with e‐cycling without assistance for EE, HR, and PO. A random effects meta‐analysis was conducted between e‐cycling with moderate assistance and walking for two outcomes, HR and VO_2_. E‐cycling with electrical assistance resulted in an increase in physiological responses assessed; however, changes were lower when compared with conventional cycling or e‐cycling with no electrical assistance. HR and VO_2_ were higher when e‐cycling compared with a walking comparison.

**FIGURE 1:**
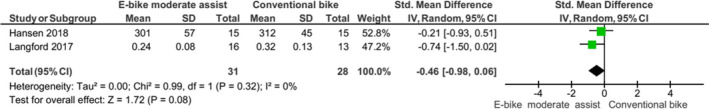
Forest plot showing the standardized mean difference in energy expenditure response when using an e‐bike with moderate electrical assistance v conventional bike

**FIGURE 2:**
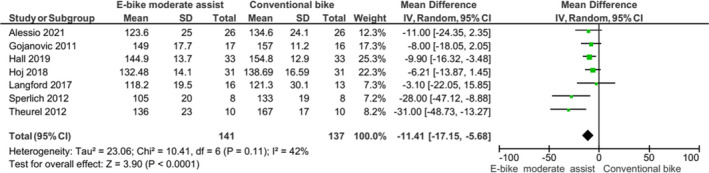
Forest plot showing mean difference in heart rate data for e‐bike with moderate electrical assistance v conventional bike

**FIGURE 3:**
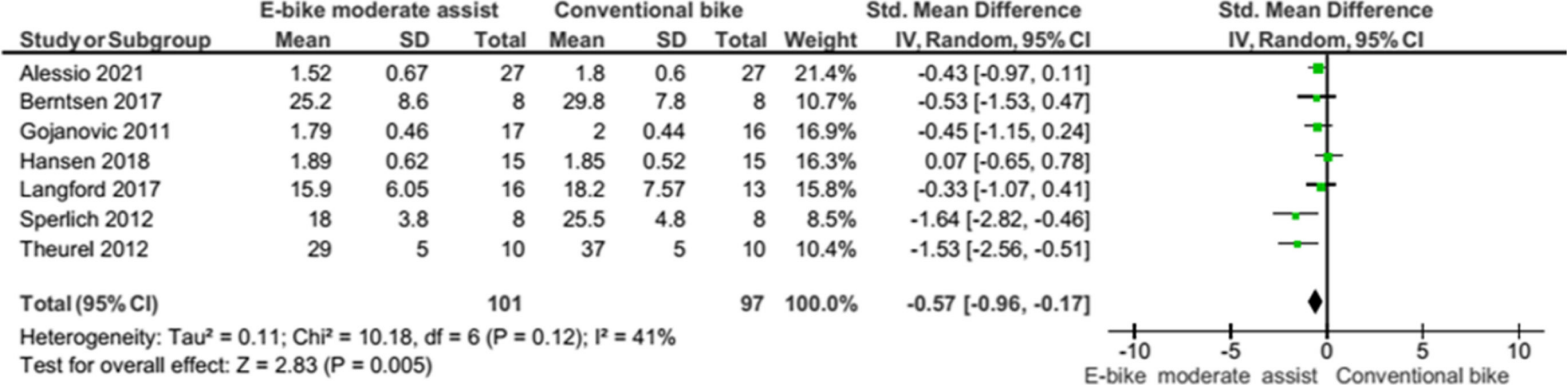
Forest plot showing oxygen uptake data for e‐bike with moderate electrical assistance v conventional bike

**FIGURE 4:**
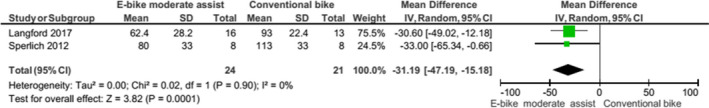
Forest plot showing mean difference in power output data for e‐bike with moderate electrical assistance v conventional bike

**FIGURE 5:**
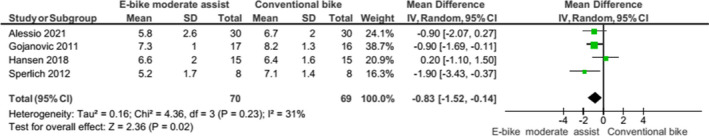
Forest plot showing mean difference in metabolic equivalent data for e‐bike with moderate electrical assistance v conventional bike

Figures 1, 2, 3, 4, 5 represent forest plots for EE, HR, VO_2_, PO & MET data compared with conventional cycling. Appendix S4 contains all forest plots.

Tables 4, 5, 6, 7, 8 represent meta‐analysis information for each outcome with comparator.

**TABLE 4:** Main effects of EE (kcal) response to e‐cycling with comparison.

E‐bike mode	Comparator	No of studies	SMD (95% CI)	*I* ^2^ (%)	*p* value
Moderate electrical assistance	Conventional bike	2	−0.46 (−0.98, 0.06)	0	0.08
Moderate electrical assistance	E‐bike no electrical assistance	3	−1.08 (−1.49, −0.66)	0	<0.00001
High electrical assistance	E‐bike no electrical assistance	2	−2.66 (−4.05, −1.28)	72	0.0002

**TABLE 5:** Main effects of HR response to e‐cycling with comparison

E‐bike mode	Comparator	No of studies	Mean difference (BPM) (95% CI)	*I* ^2^ (%)	*p* value overall
Moderate electrical assistance	Conventional bike	7	−11.41 (−17.15, −5.68)	42	<0.0001
Moderate electrical assistance	E‐bike no electrical assistance	2	−3.41 (−10.98, 4.16)	0	0.38
High electrical assistance	Conventional bike	2	−19.50 (−27.32, −11.68)	0	<0.00001
High electrical assistance	E‐bike no electrical assistance	2	−15.77 (−23.25, −8.30)	0	<0.0001
Moderate electrical assistance	Walking	2	10.38 (−1.48, 22.23)	51	0.09

**TABLE 6:** Main effects of VO_2_ response to e‐cycling with comparison

E‐bike mode	Comparator	No of studies	SMD (95% CI)	*I* ^2^ (%)	*p* value
Moderate electrical assistance	Conventional bike	7	−0.57 (−0.96, −0.17)	41	0.005
Moderate electrical assistance	E‐bike no electrical assistance	1	−0.89 (−1.86, 0.99)	53	0.08
High electrical assistance	Conventional bike	2	−1.10 (−1.56, −0.65)	0	<0.00001
Moderate electrical assistance	Walking	2	0.34 (−0.14, 0.82)	0	0.16

**TABLE 7:** Main effects of PO response to e‐cycling

E‐bike mode	Comparator	No of studies	Mean difference (W) (95% CI)	*I* ^2^ (%)	*p* value
Moderate electrical assistance	Conventional bike	2	−31.19 (−47.19, −15.18)	0	0.0001
Moderate electrical assistance	E‐bike no electrical assistance	3	−19.63 (−22.42, −16.85)	0	<0.00001
High electrical assistance	E‐bike no assistance	2	−53.71 (−64.09, −43.34)	75	<0.00001

**TABLE 8:** Main effects of MET response to e‐cycling

E‐bike mode	Comparator	No of studies	Mean difference (MET) (95% CI)	*I* ^2^ (%)	*p* value
Moderate electrical assistance	Conventional bike	4	−0.83 (−1.52, −0.14)	31	0.02

Overall, EE (kcal) increased when e‐cycling with electrical assistance; however, values were lower than any comparator; there was a small decrease in EE for e‐cycling with moderate electrical assistance compared with conventional cycling (SMD −0.46, 95% CI: −0.98, 0.06, *p *= 0.08), however, this finding was not significant. Although an increase in EE from baseline was observed during all cycling conditions, the largest difference between conditions was observed between e‐cycling with high electrical assistance and e‐cycling without electrical assistance SMD 2.66 (95% CI: −4.05, −1.28, *p *= 0.0002).

E‐cycling with electrical assistance was associated with an increase in HR response; the response was lower when compared with conventional cycling or e‐cycling without electrical assistance. E‐cycling with moderate electrical assistance resulted in a difference of −11.41 (95% CI: −17.15, −5.68) BPM compared with conventional cycling. Compared with walking, e‐cycling with electrical assistance was associated with a higher HR (MD 10.38, 95% CI: −1.48, 22.23); however, was not significant *p *= 0.09.

VO_2_ increased from baseline when e‐cycling with electrical assistance, e‐cycling without assistance, conventional cycling, and walking. VO_2_ was higher when e‐cycling with electrical assistance compared to walking; however, this finding was not significant (SMD 0.34, 95% CI: −0.14, 0.82, *p *= 0.16). VO_2_ when e‐cycling with a moderate assistance was lower compared with conventional cycling, SMD −0.57 (95% CI: −0.96, −0.17, *p *= 0.005).

PO during e‐cycling with moderate electrical assistance was lower, mean difference −31.19 W (95% CI: −47.19, −15.18 W, *p *= 0.0001) compared with conventional cycling. E‐cycling with high electrical assistance was associated with the largest difference in PO, mean difference −53.71 W (95% CI: −64.09, −43.34 W, *p *< 0.00).

A small difference is observed between e‐cycling with moderate assistance compared with conventional cycling, −0.83 METs (95% CI: −1.52, −0.14, *p *= 0.02).

## 7 DISCUSSION

In this systematic review and meta‐analysis, we aimed to quantify the physiological response from e‐cycling with electrical assistance when compared with e‐cycling without electrical assistance, conventional cycling, or walking. Overall, e‐cycling was associated with an increase in physiological responses, equivalent to moderate intensity physical activity. Across a host of physiological parameters (EE, HR, VO_2_, PO, and METs), physiological responses were lower than observed with conventional cycling, or e‐cycling without electrical assistance, but generally greater than that observed when walking. Conventional cycling, e‐cycling without assistance, and walking were used as comparators to allow for a realistic comparison of modes of active transport as conventional cycling and walking are well‐established modes of active travel. E‐cycling without assistance and e‐cycling with high assistance were included as comparators as e‐bikes without assistance are heavier than a conventional bike and people may not continuously e‐cycle with assistance on. Furthermore, people may choose to e‐cycle with a high assistance level.

## 8 STRENGTHS AND LIMITATIONS

To the best of our knowledge, this is the first meta‐analysis to quantify and compare the short‐term physiological effects of e‐cycling with more conventional forms of active transport (cycling and walking). We undertook a comprehensive search of short‐term e‐bike studies that assessed physiological responses. This study provides new data on the physiological responses associated with e‐cycling and its potential as a public health initiative for promoting recommended levels of physical activity. A limitation was the lack of data for certain outcome measures. Some results were based on two studies (HR and VO_2_ for walking comparisons), which is required by Cochrane as the minimum for a meta‐analysis; however, more studies with similar outcomes and methodologies would substantiate these findings. Moreover, topography varied between studies which could have an impact on outcomes. Our search criteria excluded non‐English manuscripts meaning studies could have been missed. Reported I^2^ values infer substantial heterogeneity between various included studies, to combat this, we ran random effects models which could have produced wider CI.

Many of the studies within this systematic review and meta‐analysis included healthy adult participants;^16^, ^19^, ^22^, ^25^, ^27^ however, it was not clear how active these adults were. Two studies recruited sedentary adults,^20^, ^24^ one study recruited both trained and untrained participants,^17^ and one study investigated a clinical population cohort, people with coronary artery disease.^15^ The heterogeneity between the participant groups may have an impact on the outcomes assessed. The quality assessment ratings of the included studies confirm the need for high quality research, future studies should aim to address these limitations.

## 9 E‐CYCLING

E‐cycling encourages people to travel further and for longer periods of time,^8^ our findings indicate the use of e‐bikes would allow people to achieve recommended levels of PA as e‐cycling can elicit moderate intensity activity levels. Our results show there is a small difference in METs between e‐cycling with moderate assistance and conventional cycling. While offering the benefits of physical activity, e‐bikes are often perceived as easier to ride and reduce concerns about cycling distance and hills.^28^ E‐bikes give riders greater control over their levels of exertion,^29^, ^30^ increase feelings of exercise self‐efficacy,^8^ and extend the active transport radius (from about 5km on a bicycle to 15km or more).^31^ Moreover, a recent study^32^ interviewed both e‐bike users and non e‐bike users and highlighted the future potential for e‐bikes to be used as an alternative to public transport, especially in a post‐COVID‐19 pandemic world. However, the interviews indicated that e‐cycling was sometimes perceived as physical inactivity;^32^ therefore, edification is required to advise this is untrue, and health benefits can be obtained from e‐cycling.

Although there is evidence to suggest e‐cycling is beneficial for health,^9^ cost is a considerable barrier for purchase of e‐bikes.^32^ To promote e‐bike use, effort should be made to make e‐bikes accessible to all individuals similar to “The eBike To Work Scheme”^33^ or e‐bike hire schemes.^34^

## 10 COMPARISONS WITH OTHER WORK

Our findings complement previous reviews that support the beneficial effects of e‐cycling on physical activity levels and health.^9^ Bourne et al.^9^ reported HR was lower when e‐cycling compared with conventional cycling, which is in agreement with our findings. VO_2_ was included within the systematic review and was reported to be lower when compared with conventional cycling or e‐cycling without support. Existing literature supports that numerous health benefits are obtained from walking.^35^ Results from walking interventions have shown increases in aerobic capacity, reductions in systolic and diastolic blood pressure, reductions in waist circumference, reductions in body fat and body mass index.^35^ Results from our meta‐analysis demonstrate an increased physiological response from e‐cycling compared with walking. Results from the meta‐analysis established e‐cycling with standard electrical support produced higher HR and VO_2_ responses compared with walking. As a result, we can infer a similar, if not increased health benefit may be ascertained from e‐cycling as an ongoing PA intervention.

## 11 FUTURE RESEARCH/IMPLICATIONS

There is a clear gap in the literature, e‐cycling should be further explored within clinical populations such as those with type 2 diabetes, pre‐diabetes, metabolic syndrome, and people who are sedentary and overweight or obese. Individuals with these metabolic conditions would benefit from increased levels of PA, associated physiological responses and health benefits,^19^ particularly as many people with these metabolic conditions face difficulties maintaining PA levels.^36^ Evidence suggests those who are inactive will benefit the most by increasing their PA levels; the health benefits are considerable for those increasing PA levels from sedentary to low levels of activity.^37^ E‐bikes offer an ideal solution and entry‐point for such populations, as they elicit the physiological responses and benefits associated with physical activity without many of the barriers associated with conventional cycling, for example, sweating and needing changing facilities,^38^ difficulty cycling uphill, and fear of cycle distance.^39^ Further research should aim to understand the best ways in which researchers and behavioral scientists can help support individuals to e‐cycle and implement e‐bikes into their day‐to‐day lives.

Future research is necessary to compare the health benefits of e‐cycling with motorized transport. By assessing the difference in cardiometabolic risk factors between those who e‐cycle regularly compared with people who use motorized transport regularly could provide a clearer understanding of the benefits of e‐cycling. Furthermore, as societies move towards greener choices,^40^ e‐cycling is an option that could be utilized due to the health and well‐being benefits previously outlined.

## 12 PERSPECTIVES

Physical inactivity (performing little or no physical activity) is an increasing problem globally. Many adults worldwide do not meet recommended guidelines for physical activity levels and therefore public health strategies are urgently needed to help people engage in regular physical activity. Research has shown that electric bikes, which provide electrical assistance while cycling may offer an ideal approach for increasing physical activity levels, particularly for those who have difficulty exercising. However, little is known about the short‐term physiological effects of electric assisted cycling (e‐cycling) and how it compares with conventional cycling. Results from this meta‐analysis showed that e‐cycling was comparable with moderate intensity physical activity, which offer health benefits. Randomized controlled trials are warranted to test this. In summary, e‐cycling offers a viable approach to support people to be more physically active. Healthcare professionals might consider encouraging e‐cycling when providing options to support people to be physically active. Moreover, government policies and public health initiatives such as subsidy of electric bikes may facilitate greater uptake.

## 13 CONCLUSION

E‐cycling was associated with an increase in physiological response that can confer health benefits. The magnitude of effect in physiological responses was lower than compared with conventional cycling. Nonetheless, e‐cycling is of sufficient intensity to meet PA recommendations. Compared with walking, e‐cycling with electrical assistance offered greater physiological response. For many people, e‐cycling offers an ideal entry approach for promotion of physical activity.

## CONFLICT OF INTEREST

The authors declare that they have no conflict of interest.

## AUTHOR CONTRIBUTIONS

JM and MK independently screened and reviewed titles and abstracts to identify eligible studies. JM, AB, and LP assessed full‐text articles for eligibility. JM and RD independently performed data extraction and quality assessment for the studies included within the meta‐analysis. JM conducted meta‐analysis. Any discrepancies faced throughout the development of the manuscript were discussed with RM. All authors contributed to the writing of manuscript. All authors read and approved final manuscript.

## Supporting information

Appendix S1

Appendix S2

Appendix S3

Appendix S4

## Data Availability

The datasets used and/or analyzed during the current study are available from the corresponding author on reasonable request.
