# Supplementary material for: Systematic review and meta‐analysis evaluating the effects electric bikes have on physiological parameters
Source: Scand J Med Sci Sports. 2022 Mar 23;32(7):1076–88. doi: 10.1111/sms.14155 (PMC9546252; doi:10.1111/sms.14155)
Supplement: Supplementary file 1 — Appendix S1 [file SMS-32-1076-s003.docx]

Search terms

Search strategy: Databases PsychINFO, MEDLINE, Embase, ISI Web of Science, CINAHL complete, SPORTDiscuss, Scopus, PubMed will be searched from inception to .

Keywords (including MeSH terms) and phrases will be incorporated. “OR” and “AND” Boolean operators will be used for within and between component/s searching, as follows:

Component 1: “Electric bike*” OR “electric bicycl*” OR “e-bike*” OR “e-cycl*” OR “e-bicycl*” OR “pedal-assist*” OR “pedelecs” OR “electric* assist* bike*” OR “electric* assist* cycl*” OR “electric* assist* bicycl*” OR “electrically-assist* bike*” OR “electrically-assist* cycl*” OR “electrically-assist bicycl*”

Component 2: “heart rate” OR “energy expenditure” OR “oxygen uptake” OR “power output” OR “work-load” OR “physiolog*” OR “metabolic” OR “exertion”

CINHAL subject headings: (MH "Heart Rate+") or (MH "Workload") or (MH "Workload Measurement") or (MH "Physiology")

Emtree (Embase subject headings): ‘heart rate’, ‘workload’, ‘cardiovascular function’, ‘physiology’

APA PsychINFO: DE "Heart Rate", DE “Physiology”

SPORTDiscuss: DE "HEART rate monitoring" OR DE “Heartbeat” OR DE “Aerobic capacity” OR DE “Cardiovascular system physiology”

IEEE Xplore: E-bike + heart rate, E-bike + energy expenditure, E-bike + oxygen uptake, E-bike + power output.
