# Supplementary material for: Systematic review and meta‐analysis evaluating the effects electric bikes have on physiological parameters
Source: Scand J Med Sci Sports. 2022 Mar 23;32(7):1076–88. doi: 10.1111/sms.14155 (PMC9546252; doi:10.1111/sms.14155)
Supplement: Supplementary file 3 — Appendix S3 [file SMS-32-1076-s004.docx]

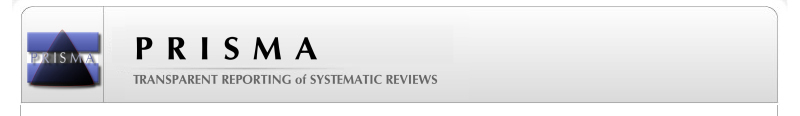
**PRISMA 2009 Flow Diagram**

Full-text articles excluded,
(n = 25)

Reasons:

Wrong study design (n = 12)

No full text available (n = 4)

Wrong outcomes (n = 4)

Full text not in English (n = 1)

Duplicate (n = 1)

Wrong comparison (n = 1)

No participants (n = 1)

Conference paper (n = 1)

Included

Eligibility

## Screeningc

Screening

## Screeningc

Screening

## Screeningc

Records excluded
(n = 589)

Studies included in quantitative synthesis (meta-analysis)
(n = 12)

Studies included in qualitative synthesis
(n = 14)

Full-text articles assessed for eligibility
(n = 39)

Records screened
(n = 628)

Records after duplicates removed
(n = 184)

Identification

## enIdentification

Additional records identified through other sources
(n = 1)

Records identified through database searching
(n =811)
