# Supplementary material for: Systematic review and meta‐analysis evaluating the effects electric bikes have on physiological parameters
Source: Scand J Med Sci Sports. 2022 Mar 23;32(7):1076–88. doi: 10.1111/sms.14155 (PMC9546252; doi:10.1111/sms.14155)

EE

Figure 1.1

E-bike moderate assistance compared with a conventional bike


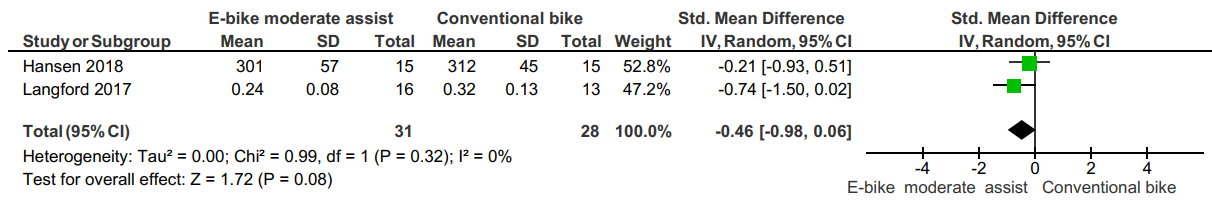


Figure 1.2

E-bike moderate assistance compared with e-bike no assistance


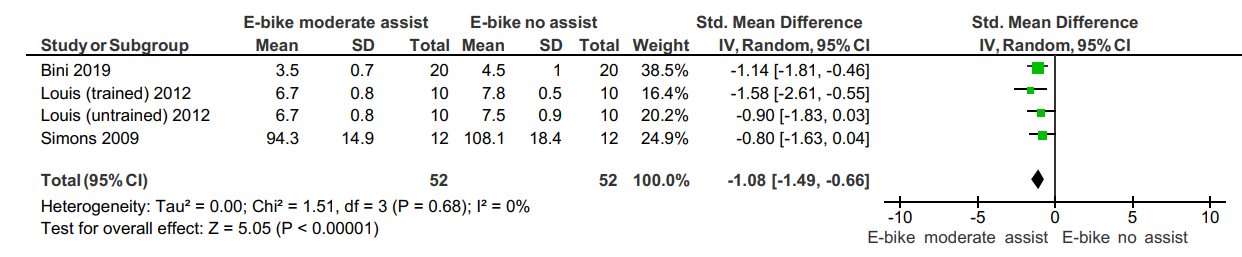


Figure 1.3

E-bike high assistance compared with e-bike no assistance


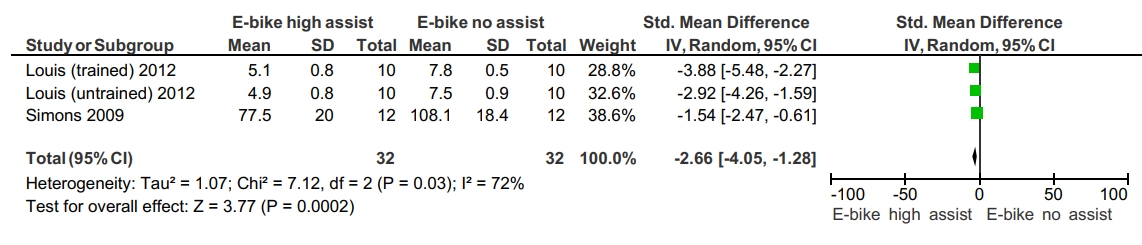


HR

Figure 2.1

E-bike moderate assistance compared with a conventional bike


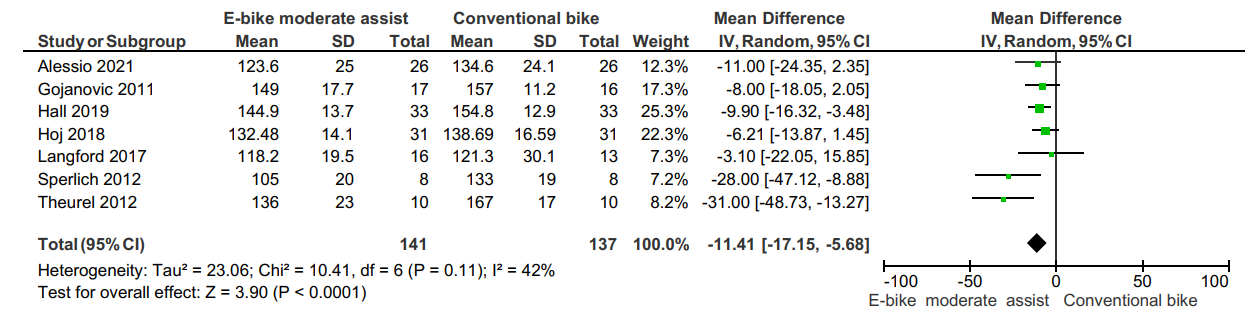


Figure 2.2

E-bike moderate assistance compared with e-bike no assistance


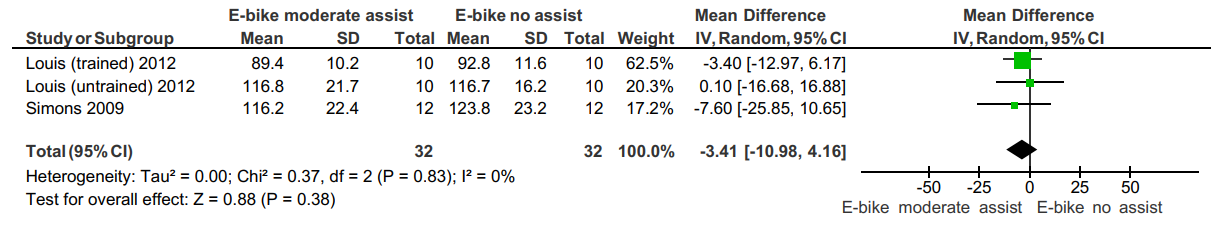


Figure 2.3

E-bike high assist compared with a conventional bike


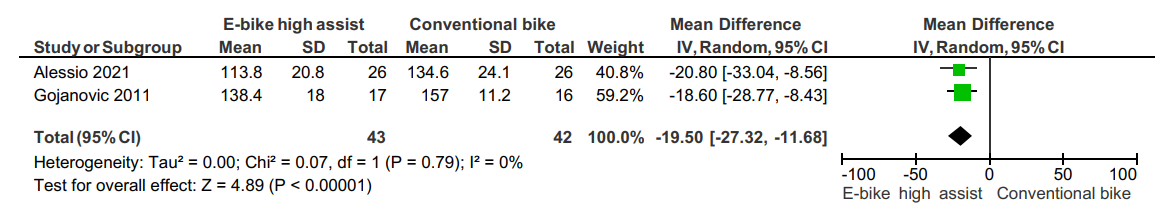


Figure 2.4

E-bike high assistance compared with e-bike no assistance


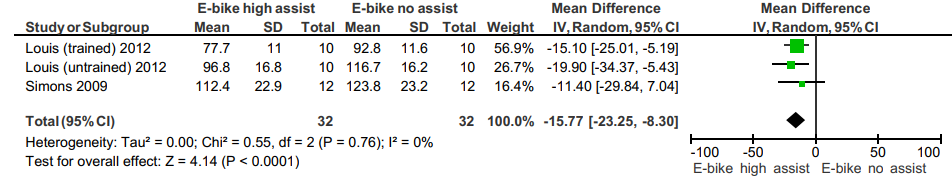


Figure 2.5

E-bike moderate assist compared with walking


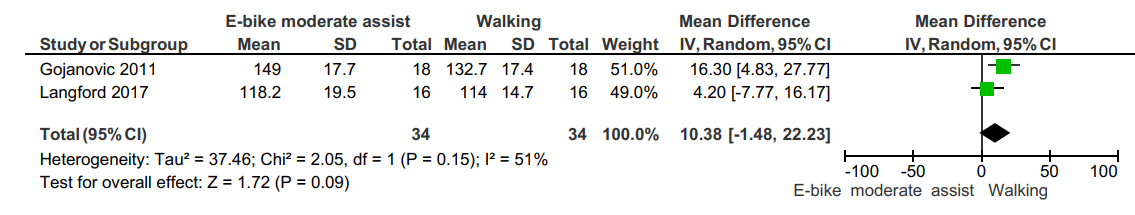


VO_2_

Figure 3.1

E-bike moderate assistance compared with a conventional bike


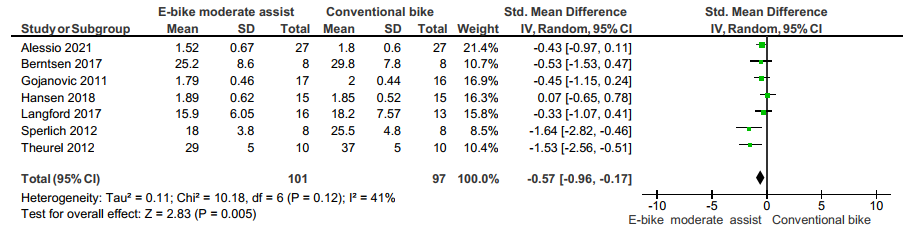


Figure 3.2

E-bike moderate assistance compared with e-bike no assistance


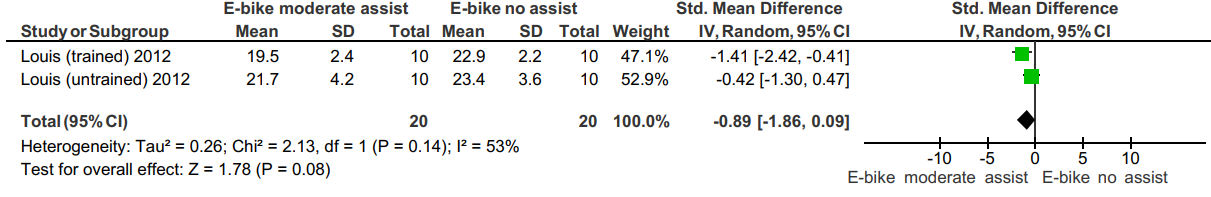


Figure 3.3

E-bike high assistance compared with a conventional bike
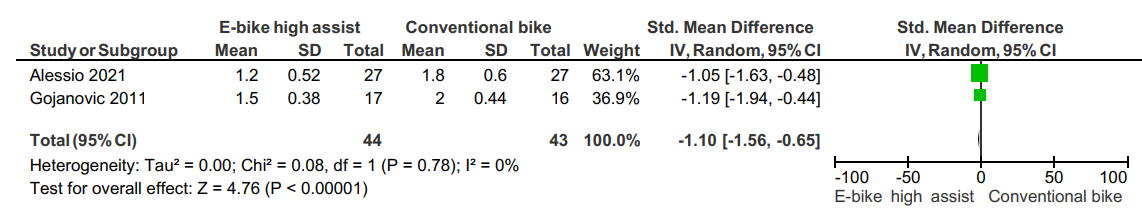


Figure 3.4

E-bike moderate assist compared with walking


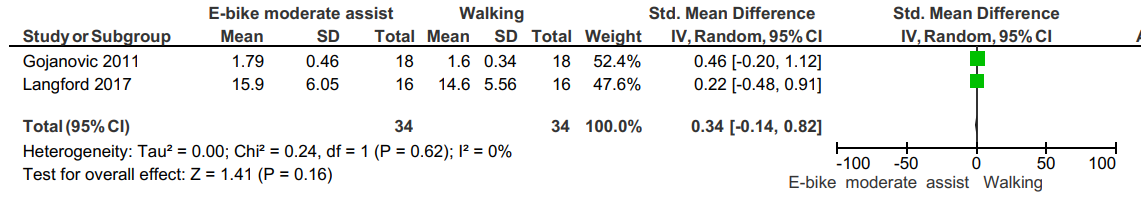


PO

Figure 4.1

E-bike moderate assistance compared with a conventional bike


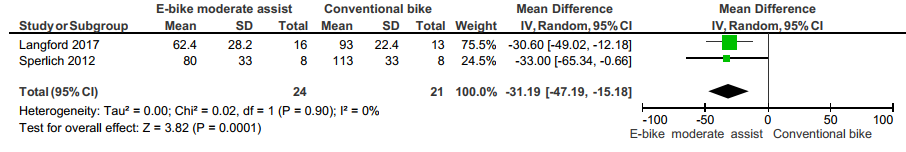


Figure 4.2

E-bike moderate assistance compared with e-bike no assistance


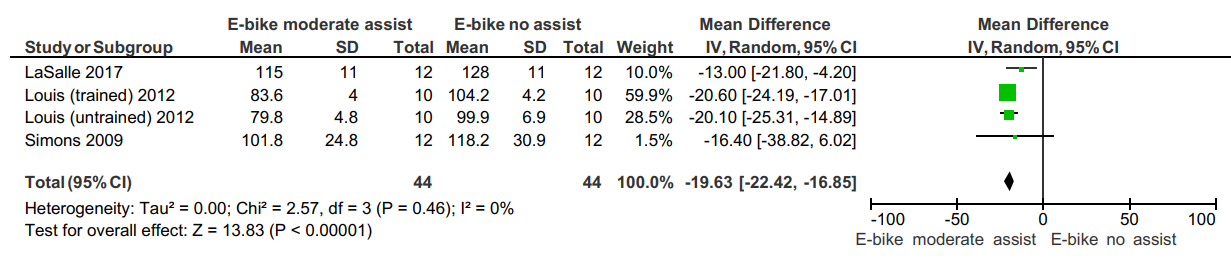


Figure 4.3

E-bike high assistance compared with e-bike no assistance


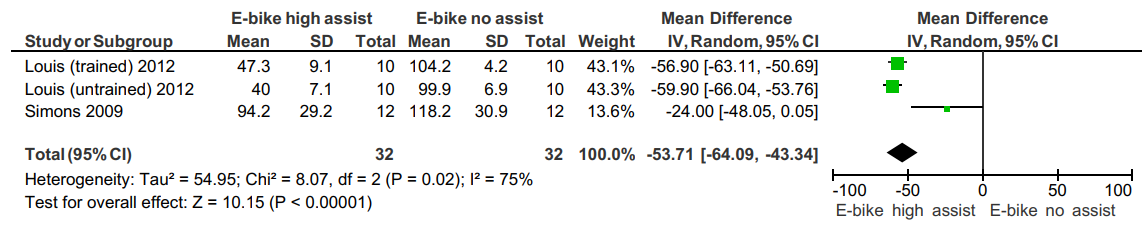


METs

Figure 5.1

E-bike moderate assistance compared with a conventional bike


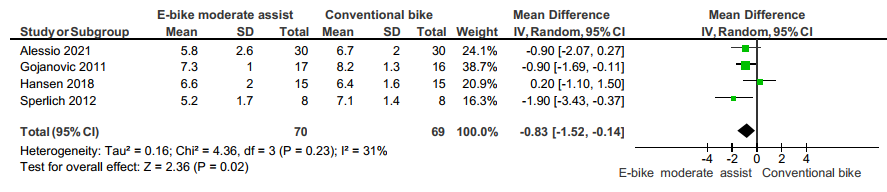

Supplement: Supplementary file 4 — Appendix S4 [file SMS-32-1076-s002.docx]
